# Full-Genome Sequence of Bacillus safensis Strain IDN1, Isolated from Commercially Available Natto in Indonesia

**DOI:** 10.1128/MRA.00180-21

**Published:** 2021-04-15

**Authors:** Masa-aki Yoshida, Masahide Uomi, Tomone Ikai, Tophil Ilado, Diana Waturangi, Januarti J. Ekaputri, Davin H. E. Setiamarga

**Affiliations:** a Oki Marine Biological Station, Faculty of Life and Environmental Science, Shimane University, Oki, Shimane, Japan; b Department of Applied Chemistry and Biochemistry, National Institute of Technology, Wakayama College, Gobo, Wakayama, Japan; c Faculty of Biotechnology, Atma Jaya Catholic University, Jakarta, Indonesia; d Department of Civil Engineering, Sepuluh Nopember Institute of Technology, Surabaya, East Java, Indonesia; Indiana University, Bloomington

## Abstract

We isolated a strain of Bacillus safensis, which we called IDN1, from natto sold in Indonesia. In order to gain insights into its genomic structure and understand its biology, we used the Oxford Nanopore MinION platform followed by PCR to verify the ends and determine its full circular genome sequence.

## Abstract (precis)

We isolated a strain of Bacillus safensis, which we called IDN1, from *natto* sold in Indonesia. In order to gain insights into its genomic structure and understand its biology, we used the Oxford Nanopore MinION platform followed by PCR to verify the ends and determine its full circular genome sequence.

## ANNOUNCEMENT

The Gram-positive, spore-forming soil bacteria of the genus *Bacillus* are known for their resilience and universal pervasiveness. Bacillus safensis was first isolated from spacecraft surfaces in space centers in the United States (1) and was thought to have possibly contaminated the surface of the planet Mars (2). In this study, we report the complete genome sequence (3,794,970 bp; GC content, 41.4%) of the *B. safensis* strain IDN1 that we previously isolated from the Japanese traditional fermented food natto, which was sold in a market in Indonesia (3). A single colony was picked from the glycerol stock of the isolated strain and then cultured in LB broth overnight at 37°C. Genomic DNA was extracted using the Qiagen DNeasy isolation kit, following the manufacturer’s instructions. After isolation, we amplified the 16S rRNA for species identification, for which we obtained a very good match with both Bacillus safensis and Bacillus pumilus (sequence similarity of >99% to GenBank accession number KT937148.1; E value of 0.0; as of August 2020 by NCBI Web BLAST). The lack of nucleotide substitutions in the 16S rRNA gene sequence to differentiate the two species has been well recorded (4). To decisively identify the strain, we also sequenced *gyrB* because previous studies have shown that the gene is useful for differentiating *B. safensis* and *B. pumilus* (1, 4). The BLASTn result of *gyrB* clearly showed that IDN1 was a strain of *B. safensis* (sequence similarity of 99% to GenBank accession number JX183212.1; E value of 0.0; as of August 2020 by NCBI Web BLAST) (5). To sequence the full genome of Bacillus safensis strain IDN1, we first prepared sequencing libraries from the extracted, nonfragmented bacterial genomic DNA (ca. 400 ng) using the SQK-RAD004 rapid sequencing kit (Oxford Nanopore Technologies [ONT]). Long-read sequencing was performed using the MinION R9.5.1 flow cell (FLO-MIN107). Sequencing quality was monitored from the MinKNOW interface release 19.06.7 (ONT). Sequences were base called using Guppy v3.1.5. Default parameters were used for all software unless otherwise specified. A total of 1,228,618 reads (3,148,033,402 bp) were obtained, with an average length of 2,562 bp. For the assembly process, a total of 1.0 Gbp of reads were quality filtered and subsampled by Filtlong v0.2.0 (https://github.com/rrwick/Filtlong) to filter by lengths of 1,000 bp (-min_length 1000) and quality of 1 (-min_mean_q 80), resulting in around 200× expected coverage. The subsampled reads were assembled with Canu v1.8 (6) into a single linear contig. Assembly errors were checked and corrected four times using Racon v1.4.0 (7) with all reads. To verify the circularity of the genome, first, we searched for reads containing sequences of both ends of the linear contig, for which we found 19 reads. The reads aligned well with each other, and no gap was found at the end of the contig. PCR and Sanger sequencing performed using primers designed at both ends of the contig (forward primer, GTCGCGGAAGCACCAGCAAA; reverse primer, GAAGTCGACAGCTTTGTTATCT) also verified that the obtained draft genome was circular with the ends closed without any gap. Annotation was performed using DFAST v1.1.0 from the DDBJ (8). The total length of the genome is 3,794,970 bp, with a GC content of 41.4%. DFAST predicted 7,246 protein-coding sequences, 24 rRNA genes, and 79 tRNA genes. A single copy of the beta carbonic anhydrase (*ca*) gene was found in the genome (5, 9). This agrees with previous reports suggesting the availability of this gene in the genus *Bacillus* (10–12), and in this strain, as suggested by a PCR survey of the genome using *ca*-specific primers (5). The presence of this gene might explain the biomineralizing ability of this strain, as suggested previously (3). The genes alcohol dehydrogenase (*adh*), aldehyde dehydrogenase (*aldh*), and cytochrome P450 monooxygenase (*cyp*) were also found in the genome. The genes are known to be involved in hydrocarbon decompositions and present in various crude oil-degrading bacteria, such as Franconibacter pulveris (13), although further study is still needed to confirm if IDN1 also has such capability. We also found the C4-dicarboxylate transporting protein (DctA), which was 97% identical to a dicarboxylate-transporting protein found in several other strains of *B. safensis* (e.g., GenBank accession number WP_041107478.1) (14). This suggests a possible usefulness of IDN1 for bioremediation of aldehyde-contaminated environments. We believe the information provided in this manuscript will be useful for further dissections of the biological nature of this strain. The genomic information of IDN1 will also contribute insights into possible industrial utilizations of this strain, other than for producing natto.

### Data availability.

An assembled bacterial chromosome draft sequence was deposited in DDBJ under the accession number AP021906. Raw ONT reads are available in the DDBJ Sequence Read Archive (DRA) under BioProject accession number PRJDB8990 and DRA accession number DRA009244.
